# DNA damage in mitosis: SOD1 delays anaphase onset

**DOI:** 10.1111/febs.70264

**Published:** 2025-09-12

**Authors:** George Zachos

**Affiliations:** ^1^ Department of Biology University of Crete Heraklion Greece

**Keywords:** anaphase, DNA damage, kinetochores, mitosis, phosphatase, PP2a, SOD1

## Abstract

Unrepaired DNA double strand breaks (DSBs) can lead to genomic instability, carcinogenesis, or cell death; however, mitotic cells do not exhibit a DNA damage checkpoint delay and do not repair DSBs until the next cell cycle. Instead, DSBs can delay anaphase through the mitotic spindle checkpoint by an incompletely understood mechanism. Li *et al.* now show that, in human mitotic cells with damaged DNA, superoxide dismutase 1 inhibits protein phosphatase 2a, which dephosphorylates kinetochore proteins to silence the spindle checkpoint, leading to persistent spindle checkpoint activation and delayed anaphase onset. Here, the biological significance of these findings and open questions are discussed.

## Graphical abstract

Mitotic cells do not exhibit a DNA damage checkpoint delay and do not repair DNA double strand breaks (DSBs) until the next cell cycle. Instead, DSBs can delay anaphase through the mitotic spindle checkpoint, by an incompletely understood mechanism. Li *et al.* now show that, in human mitotic cells with damaged DNA, SOD1 inhibits PP2a, which dephosphorylates kinetochore proteins to silence the mitotic spindle checkpoint, leading to persistent spindle checkpoint activation and delayed anaphase onset.

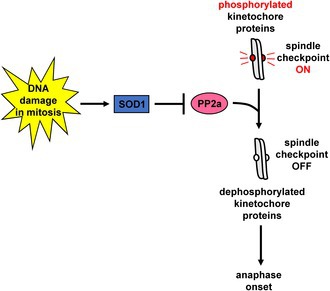

**Abbreviations**
53BP1: p53‐binding protein 1
ATM: Ataxia‐telangiectasia mutated
ATR: Ataxia telangiectasia and Rad3‐related
Cdk: Cyclin‐dependent kinase
DSB: DNA double strand break
KMN: Knl1, Mis12, and Ndc80
MRN: Mre11‐Rad50‐Nbs1
PP2a: Protein Phospatase 2a
SOD1: Superoxide dismutase 1

## Introduction

DNA double strand breaks (DSBs) are one of the most dangerous types of DNA lesions. If unattended, they can lead to genome rearrangements, genomic instability, oncogenic transformation, or cell death; as a result, interphase cells respond to DSBs by activating DNA damage checkpoints; that is, cell surveillance mechanisms that delay cell cycle progression until the DNA lesions are repaired [1]. The ataxia‐telangiectasia mutated (ATM)‐Chk2 and the ataxia telangiectasia and Rad3‐related (ATR)‐Chk1 are the main DNA damage checkpoint signaling pathways in vertebrate cells [1]. In response to DSBs in interphase, the ATM and ATR kinases are recruited to the DNA damage sites through interaction with various adaptor complexes and partner proteins. There, ATM/ATR phosphorylate several targets including the downstream effector kinases Chk1/Chk2; in turn, phosphorylated (active) Chk1/Chk2 prevent activation of cyclin‐dependent kinases (Cdks) to delay cell‐cycle progression (reviewed in [1]).

However, after cells enter mitosis and move past prophase, there is no DNA damage checkpoint delay, and DNA is not repaired until daughter cells enter the G_1_ phase of the next cell cycle [2, 3]. In the presence of DSBs, mitotic cells activate early DNA damage signaling events such as phosphorylation of histone variant γ‐H2AX (a DNA damage‐associated histone mark) and recruitment of the Mre11‐Rad50‐Nbs1 (MRN) adaptor protein complex to the DNA damage sites; however, downstream events such as accumulation of p53‐binding protein 1 (53BP1), which facilitates DSB signaling and repair, or Chk1/Chk2 phosphorylation are blocked [4]. Why the DNA damage response is “truncated” in mitosis is incompletely understood: For example, it is unclear whether this partial response contributes to genomic stability or happens out of necessity because several DNA damage response proteins are rewired in mitosis to assist with the complex task of chromosome segregation [5]. As a result, mitotic cells “mark” the DNA damage sites, “tether” broken chromosome ends until they can be repaired in the following G_1_ phase, and proceed with chromosome segregation [6, 7]. Despite this, relatively high levels of DSBs can still delay transition from metaphase to anaphase; however, this is due to activation of the mitotic spindle checkpoint, rather than a result of DNA damage response signaling, by an incompletely understood mechanism [8].

The mitotic spindle checkpoint delays onset of anaphase until all sister kinetochores are bipolarly attached to spindle microtubules; as a result, this mechanism provides cells with more time to correct misattached kinetochore‐microtubules to avoid possible chromosome segregation errors [9]. In response to unattached kinetochores, conserved spindle checkpoint components such as the Mad (Mad1, Mad2, and BubR1) and Bub (Bub1 and Bub3) proteins accumulate to the outer kinetochore region, facilitated by the KMN (Knl1, Mis12, and Ndc80) protein network, and this accumulation is essential to prevent activation of the anaphase‐promoting complex/cyclosome to delay mitotic exit [9]. The mitotic kinase Mps1 phosphorylates Knl1 at the conserved MELT repeats (named after the single amino acid code), and this phosphorylation is essential for recruitment of Bub and Mad proteins to unattached kinetochores [9]. Furthermore, phosphorylation of BubR1 by mitotic kinases promotes the recruitment of protein phosphatase 2a (PP2a), which opposes Mps1‐mediated phosphorylation of Knl1, generating a negative feedback loop that can rapidly switch the mitotic spindle checkpoint off when (stable) bipolar kinetochore‐microtubule attachments are formed [10, 11]. However, how DSBs promote spindle checkpoint signaling to delay mitotic progression remains unclear. Li *et al*. now offer new light on this long‐standing issue by identifying superoxide dismutase 1 (SOD1) as a novel regulator of the DNA damage‐induced anaphase delay in human cells [12].

## SOD1 is a previously undescribed protein that is required for anaphase delay in response to DNA damage

By investigating human cell lines by live‐cell microscopy, Li *et al*. show that treatment of cells with DNA damaging agents increases the time cells spent in mitosis from cell rounding until anaphase onset, from approximately 52 min in untreated cells to around 62 min after DNA damage, in agreement with previous findings [8]. To identify previously undescribed proteins that regulate the DNA damage‐induced anaphase delay, they performed a high‐throughput siRNA screen measuring the amount of cells expressing the mitotic marker phospho‐histone H3 after exposure to ionizing radiation by flow cytometry, followed by a secondary screen measuring the mitotic transit time by live‐cell microscopy. Remarkably, Li *et al*. identify SOD1 as a novel protein whose depletion impairs the anaphase delay in response to ionizing radiation [12].

## SOD1 delays mitotic progression by inhibiting PP2a phosphatase

**Fig. 1:**
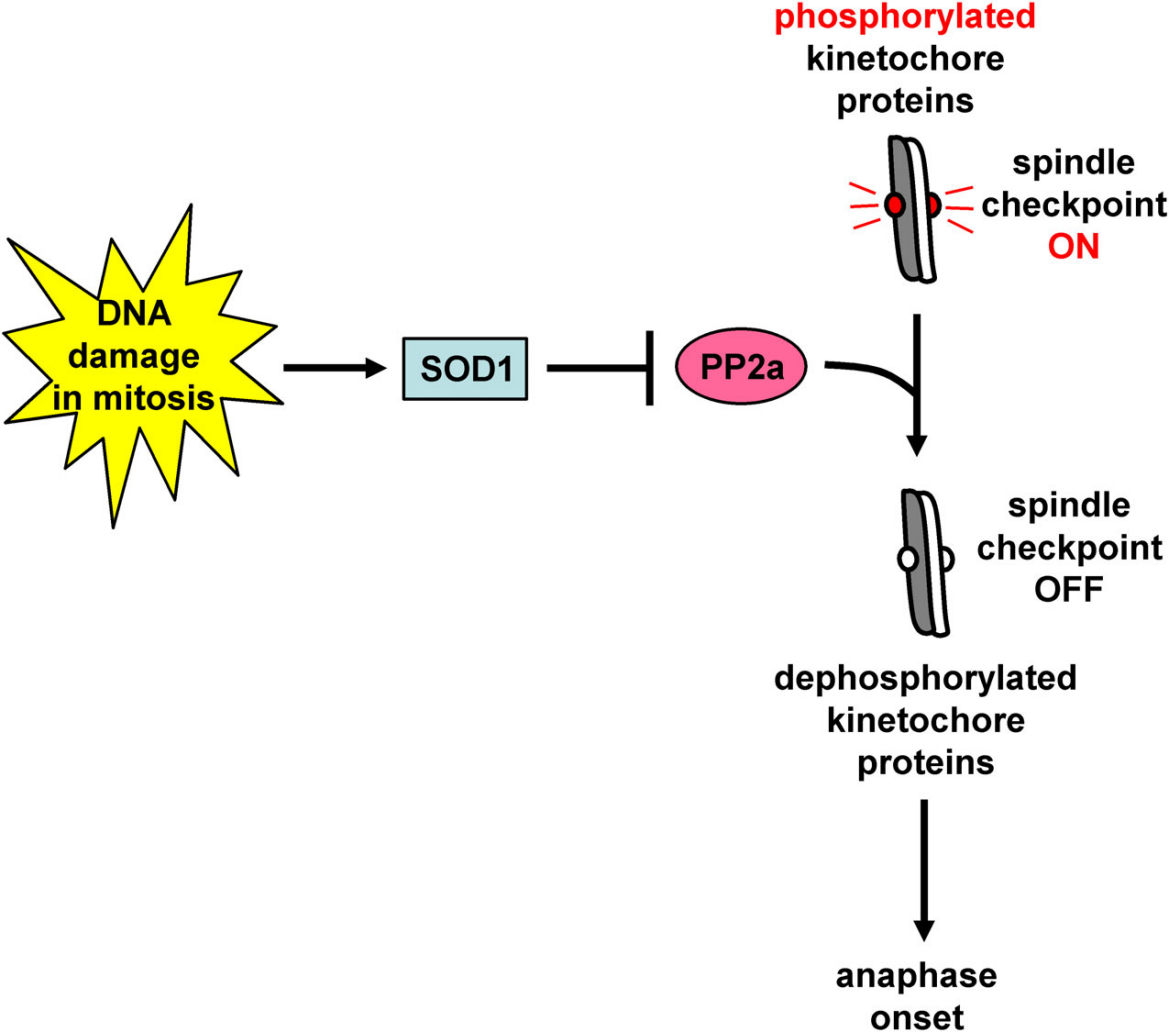
Model for the mechanism by which superoxide dismutase 1 (SOD1) delays anaphase onset in the presence of DNA damage. In mitotic cells with damaged DNA, SOD1 inhibits protein phosphatase 2a phosphatase activity, which is required for dephosphorylation of kinetochore proteins and spindle checkpoint silencing, to delay the onset of anaphase.

SOD1 is an abundant Cu/Zn enzyme found in the cytoplasm and mitochondria, which converts superoxide radicals into hydrogen peroxide (H_2_O_2_) and molecular oxygen, thus protecting cells from the harmful consequences of these reactive oxygen species [13]. By depleting HeLa cells of SOD1 by siRNA or analyzing paired HeLa wild‐type and SOD1‐knockout cells, Li *et al*. show that SOD1 is required for anaphase delay after treatment with ionizing radiation, regardless of whether DNA damage occurred in mitosis or interphase [12]. SOD1‐depletion correlates with increased PP2a phosphatase activity in cell extracts; furthermore, pharmacological inhibition of PP2a rescues mitotic delay in SOD1‐deficient cells, suggesting that SOD1 inhibits PP2a to delay anaphase onset in response to damaged DNA [12]. DNA damage also induces phosphorylation of BubR1 and Knl1‐MELT repeats at prometaphase kinetochores in wild‐type cells compared with controls in unperturbed mitosis; furthermore, these phosphorylation events are diminished in SOD1‐depleted cells, suggesting that SOD1 is required for persistent spindle checkpoint activation in response to damaged DNA [12]. Impaired anaphase delay in SOD1‐deficient cells correlates with increased micronuclei formation, indicating mis‐segregated chromosomes or chromosome fragments, and 53BP1 foci after mitotic exit compared with controls [12]. However, SOD1‐depletion does not accelerate mitotic exit in unperturbed mitosis, or after treatment of cells with the microtubule poison nocodazole that generates unattached kinetochores, indicating that SOD1 is not required for the canonical spindle checkpoint response, that is, in the absence of DNA damage [12]. On the basis of those findings, the following model is proposed: In the presence of DNA damage, SOD1 inhibits PP2a phosphatase activity, which is required for dephosphorylation of key kinetochore proteins and spindle checkpoint silencing, resulting in prolonged spindle checkpoint activation and delayed onset of anaphase (Fig. 1).

## Conclusions and open questions

The results by Li *et al*. identify a previously undescribed regulator of the DNA damage‐induced mitotic delay in human cells and also raise several important questions: For example, how is SOD1 activated specifically by DNA damage? Does SOD1 localize to kinetochores or does it function in the cytosol? And how does SOD1 inhibit PP2a catalytic activity? It was previously proposed that DNA damage induces reactive oxygen species, at least in part through phosphorylation of γ‐H2AX [14]. Furthermore, SOD1 can inactivate phosphatases in growth factor signaling cascades, perhaps by catalyzing the production of H_2_O_2_, leading to oxidization of cysteine residues in the phosphatase's active site [15]. Therefore, one possibility is that phosphorylation of γ‐H2AX after DNA damage leads to generation of reactive oxygen species, which are converted into H_2_O_2_ by SOD1, causing local inactivation of PP2a phosphatase. This could explain why DNA damage‐induced mitotic delay is dose‐dependent [8, 12], because higher levels of DNA damage could lead to increased amounts of SOD1‐generated H_2_O_2_ thus inhibiting more PP2a molecules. This would also predict that SOD1 does not inhibit PP2a in the absence of DNA damage when the amount of reactive oxygen species is relatively low, in agreement with the findings by Li *et al*. [12].

Also, what is the mechanism by which PP2a inhibits anaphase onset in response to DNA damage? In unperturbed mitosis, after stable kinetochore‐microtubule attachments are established, PP2a opposes local phosphorylation of Knl1 by Mps1 kinase, leading to the removal of BubR1 and Mad1/Mad2 complexes from kinetochores and spindle checkpoint silencing [10, 11]. It is therefore important to examine whether a similar mechanism operates in the presence of damaged DNA. Finally, what is the role of the SOD1‐induced anaphase delay? Although this delay is relatively modest compared with the spindle checkpoint delay by microtubule poisons or the DNA damage checkpoint delay, which can last for several hours, it may provide the cell with just enough time, for example, to tether broken chromosome ends to segregate them correctly. Consistently, SOD1‐deficient cells exhibit increased frequency of micronuclei after ionizing radiation treatment compared with controls, by fluorescence microscopy analysis of fixed samples [12]. Further analysis using long‐term live‐cell microscopy will help us better understand the biological significance of DNA damage‐induced mitotic delay for chromosome stability, cell proliferation, and survival.

## Conflict of interest

The author declares no conflict of interest.
